# Host–Parasite Interactions and the Evolution of Gene Expression

**DOI:** 10.1371/journal.pbio.0030203

**Published:** 2005-05-31

**Authors:** Scott L Nuismer, Sarah P Otto

**Affiliations:** **1** Department of Biological Sciences, University of Idaho, Moscow, Idaho, United States of America,; **2** Department of Zoology, University of British Columbia, Vancouver, British Columbia, Canada; University of North CarolinaUnited States of America

## Abstract

Interactions between hosts and parasites provide an ongoing source of selection that promotes the evolution of a variety of features in the interacting species. Here, we use a genetically explicit mathematical model to explore how patterns of gene expression evolve at genetic loci responsible for host resistance and parasite infection. Our results reveal the striking yet intuitive conclusion that gene expression should evolve along very different trajectories in the two interacting species. Specifically, host resistance loci should frequently evolve to co-express alleles, whereas parasite infection loci should evolve to express only a single allele. This result arises because hosts that co-express resistance alleles are able to recognize and clear a greater diversity of parasite genotypes. By the same token, parasites that co-express antigen or elicitor alleles are more likely to be recognized and cleared by the host, and this favours the expression of only a single allele. Our model provides testable predictions that can help interpret accumulating data on expression levels for genes relevant to host−parasite interactions.

## Abstract 2

A model reveals that hosts should evolve co-expression of resistance alleles to recognize a range of parasites, but the parasite shouldn't evolve co-expression of infection alleles because it enhances recognition by the host.

## Introduction

Hosts and parasites are locked in a continual co-evolutionary race, which generates persistent selection for resistant hosts and infectious parasites. Understanding the direct effects of this process on spatial patterns of local adaptation [1–5 ], the evolution of virulence/pathogenicity [6–8 ], and the spread of infectious disease [9–11 ] has been a central focus of research into host−parasite interactions. Yet host−parasite interactions also generate indirect selection on a variety of other features of the interacting species. The classical example of indirect selection imposed by host−parasite interactions is on the mode of reproduction [12–16]. Host−parasite interactions can select for sexual rather than asexual reproduction, although they tend to do so only when selection is strong and sex is rare [17]. Recently, we have shown that indirect selection also acts on genome size (ploidy level), with selection favouring diploidy more often among host species and haploidy more often among parasite species [ 18]. There are a variety of other genomic features besides ploidy level that should experience indirect selection in response to host−parasite interactions. Here, we examine the evolution of expression levels using a model that is structurally similar to models of the evolution of dominance (as in the classic papers by Fisher [19, 20], Wright [21–23], and Haldane [24, 25], and more recent papers reviewed in Otto and Bourguet [26 ]).

### The Model

To explore the evolution of expression levels, we assumed that infection/resistance was determined by a single gene in the host with alleles *A* and *a,* and a single gene in the parasite with alleles *B* and *b*. We then tracked changes in allele frequency at a single modifier locus, whose alleles *(M* and *m)* altered the pattern of expression in heterozygotes at the A locus (if in hosts) or B locus (if in parasites). Thus, we refer to this modifier locus, M, as a regulatory locus. To simplify the analysis and interpretation, we allowed expression levels to evolve in only one species (the “focal species”) at a time.

**Figure 1:**
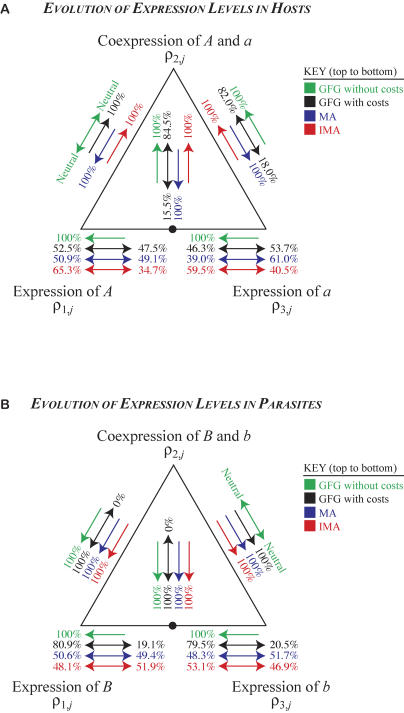
Expression Levels Are Allowed to Evolve toward Any Point in the Triangle For example, the circle corresponds to the additive case, where heterozygotes are equally likely to express either *A* only or *a* only and so have fitness halfway between the fitnesses of *AA* and *aa* individuals. The evolution of expression levels predicted by the quasi-linkage equilibrium analysis is indicated by the direction of arrows. Double-headed arrows indicate that the quasi-linkage equilibrium analysis predicts an outcome that depends on allele frequencies. Results from numerical simulations are shown as percentages of total parameter combinations that resulted in evolution of expression levels in the direction shown. Entries labelled “neutral” are cases where no change in modifier frequency occured. The range of parameter values used in these simulations is described in the main text. Predicted patterns for the host are shown in (A), and those for the parasite are shown in (B).

Determining how expression patterns evolve during the course of host−parasite co-evolution requires that we relate expression patterns to the phenotype expressed by heterozygous genotypes. We assumed that a heterozygous individual of species *j* could express the phenotype of homozygotes carrying allele *A* (or *B*) with probability *ρ*_1,*j*_ *,* *A* and *a* (or *B* and *b*) with probability *ρ*_2,*j*_ *,* and *a* (or *b*) with probability *ρ*_3,*j*_ *,* where the terms in parentheses are appropriate when the focal species is the parasite. These probabilities were assumed to sum to one (*ρ*_1,*j*_ + *ρ*_2,*j*_ + *ρ*_3,*j*_ = 1), for both hosts *(j* = *h)* and parasites *(j* = *p)*. This constraint prevents heterozygotes from having fitness greater than the best homozygous genotype in any given encounter between host and parasite genotypes. An implicit assumption of this mapping between genotype and phenotype is that heterozygotes can, if *ρ*_2_ = 1, co-express both alleles without decreasing the function of either allele. To take a concrete example, our mapping of phenotype onto genotype assumes that *Aa* hosts could express receptor *A* as effectively as *AA* hosts and also express receptor *a* as effectively as *aa* hosts. The model is easily generalized, however, to relax this assumption (results available upon request). Alleles at the regulatory locus, M, were allowed to alter the pattern of expression in heterozygotes by altering the probabilities, *ρ_i_*_,*j*_. Because an individual's genotype at the regulatory locus determines these probabilities, we specify the genotype in square brackets (e.g., *ρ_i,j_*[*MM*]). When exposed to selection induced by the interacting species, alleles at the regulatory locus might evolve to upregulate one allele over the other or to express both alleles equally (co-expression), as illustrated in Figure 1.

We incorporated host−parasite co-evolution into the modifier framework described above by considering the following well-studied genetic interactions. In the gene-for-gene (GFG) model [27], avirulence alleles in the parasite produce signal molecules that elicit a defence response in resistant hosts, whereas parasites carrying virulence alleles fail to produce the signal molecule and cannot be detected by any host. GFG interactions are considered to be prevalent in plant–pathogen interactions [28]. Costs of resistance and virulence alleles have been demonstrated in some GFG systems [29,30], so we let *C_h_* be the fitness cost of expressing only the resistant allele in hosts, and *C_p_* be the fitness cost of expressing only the virulence allele in parasites. Co-expressing both alleles might reduce these costs, particularly when the susceptible allele in the host or the avirulent allele in the parasite performs a beneficial function. The fitness costs experienced by heterozygotes expressing both alleles were thus set to *c_h_* in hosts and *c_p_* in parasites. The matching-alleles (MA) model is predicated upon a system of self/non-self recognition. Hosts can successfully defend against attack by a parasite whose genotype does not match their own. Such recognition systems have been observed in invertebrates [31] and vertebrates [32]. Finally, in the inverse-matching-alleles (IMA) model, host defence involves an array of recognition molecules (e.g., antibodies) that are able to recognize specific antigens and resist attack by parasites carrying these antigens [32]. Following the rules imposed by each of these modes of co-evolution allowed us to create a matrix that describes the outcome of an interaction between any two phenotypes (Table 1). In all cases, we assumed that infection results in a loss of host fitness but an increase in parasite fitness.

**Table 1:**
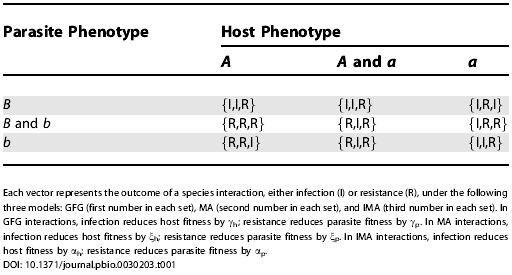
An Interaction between a Host and a Parasite Results in Either Infection or Resistance, Depending on the Phenotype of the Interacting Species

We assumed a life cycle where selection due to interactions between host and parasite was followed by sexual reproduction. Species interactions are assumed to depend on loci: a regulatory locus with alleles *M* and *m* and an interaction locus with alleles *A* and *a* if the focal species is the host, or *B* and *b* if the focal species is the parasite. Thus, there are four chromosome types in each species: *MA (MB), Ma (Mb), mA (mB),* and *ma (mb),* where the terms in parentheses correspond to cases where the focal species is the parasite. We track evolution at the regulatory locus in only one species at a time and assume that the regulatory locus is fixed on *M* in the non-focal species. The non-focal species is assumed to be diploid, although results derived with a haploid non-focal species were similar. Species *j* is assumed to undergo sexual reproduction with random mating with probability *sex_j_* and to reproduce asexually with probability (1 − *sex_j_*). During sexual reproduction, the two loci are allowed to recombine at rate *r_j_*.

Genotype frequencies after one round of selection can be determined using standard population genetic equations once the fitnesses of genotypes have been determined. We assume that encounters between species occur at random and that at most one interaction occurs per generation per individual. When interacting with genotype *k* in species *j¯* *,* the fitness of genotype *i* in species *j* is denoted by *W*_*i.j*↔*k.j¯*_ *,* where *j* = *h* and *j¯* = *p* when the focal species is a host, and *j* = *p* and *j¯* = *h* when the focal species is a parasite. The average fitness of genotype *i* in species *j* is given by its fitness in the presence of genotype *k* in the interacting species, weighted by the frequency of genotype *k,* summed over all *k:*



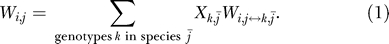



Thus, we assume that fitness depends upon genotype frequencies but is independent of the population sizes of the interacting species (e.g., [33,34]). The mean fitness in species *j* is calculated as the weighted sum of equation 1 over all genotypes in species *j:*



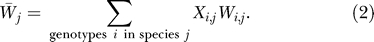



*W*_*i.j*↔*k.j¯*_ can be calculated using Table 1 and the probabilities, *ρ_i,j_,* that heterozygous hosts (parasites) express a particular phenotype. The encounter rate between hosts and parasites is implicitly incorporated in *W*_*i.j*↔*k.j¯*_ *;* when hosts and parasites rarely encounter one another, the fitnesses will be more similar to one another, all else being equal.

Assuming an infinite population size and ignoring mutation, we can write down recursions for the frequency, *X_i,j_,* of each diploid genotype (e.g., *i* = *MA*/*Ma* or *MB/Mb*) in species *j* after one round of selection followed by reproduction. For example, the first four recursions for host genotypes are given by



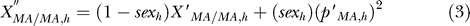





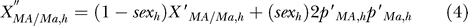





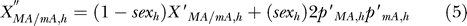





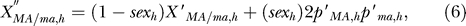



where primes indicate post-selection genotype *(X′_i,j_)* and gamete *(p′_i,j_)* frequencies. Specifically, the frequency of genotype *i* after selection is given by *X′_i,j_* = *X_i,j_* *W_i,j_*/*W¯_j_,* and the gametes produced by the surviving hosts are in the following frequencies:



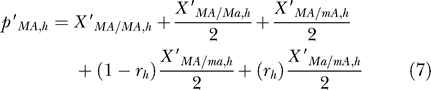





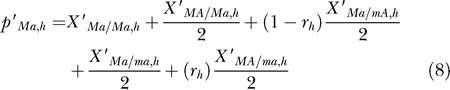





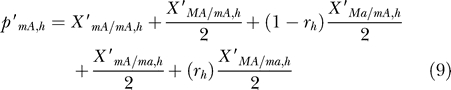





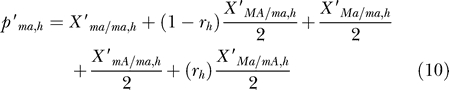



Recursions for the parasite species are identical, with the exceptions of the subscripts *A, a,* and *h,* which are replaced by *B, b,* and *p,* respectively.

## Results

To analyze the model, we assumed that selection was weak relative to the rate of recombination between the modifier locus and the locus determining infection/resistance. This allowed us to derive very general conditions for the evolution of expression levels in the focal species using quasi-linkage equilibrium approximations [35,36]. In short, the frequency of sex and recombination are assumed to be high enough relative to the strength of selection that the disequilibrium between the regulatory and interaction locus (*D_h_* = freq(*MA*)_*h*_freq(*ma*)_*h*_ − freq(*Ma*)_*h*_freq(*mA*)_*h*_ in hosts) reaches a steady-state value that depends on the current allele frequencies in the host and parasite. Solving for this disequilibrium then allows us to calculate the rate of allele frequency change at the regulatory locus to leading order in the selection coefficients (Protocol S1).

When the host was the focal species, the frequency of allele *M* at the regulatory locus changed at a per-generation rate of:



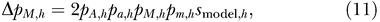



where *p_i_*_,*j*_ is the frequency of allele *i* in species *j* and where *s*_model,*h*_ depends on the model of host−parasite interactions and, for the GFG, MA, and IMA models, is given by



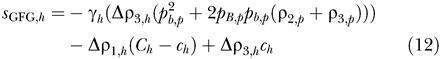





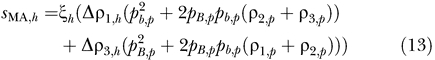





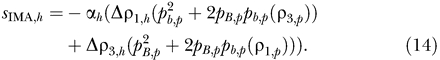



In equations 12–14, γ*_i_,* ξ*_i_,* and α*_i_* measure the strength of selection acting on species *i* due to GFG interactions, MA interactions, and IMA interactions, respectively (see Table 1), and Δ*ρ_i_*_,*j*_ represents the average effect of allele *M* on the probability of expression pattern *i* in species *j:*



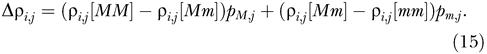



Assuming weak selection, equation 11 is equivalent to the allele frequency change in a standard one-locus model with a selection coefficient given by the selection term *s*_model,*h*_ multiplied by the frequency of *Aa* hosts (2*p_A,h_p_a,h_*). As discussed in greater detail in Protocol S1, the selection coefficient *s*_model,*h*_ can be readily interpreted on the basis of how changes in expression pattern alter the likelihood that a heterozygous host will be infected.

Similarly, when the parasite was the focal species, the frequency of allele *M* at the regulatory locus changed at a per-generation rate of



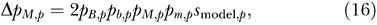



where now



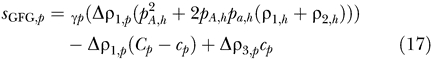





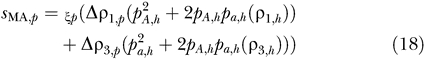





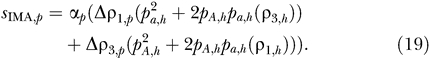



To the order of these approximations, genetic associations *(D_h_, D_p_)* had no influence on the frequency of the alleles at the regulatory locus, M. Instead, frequency change at the regulatory locus resulted from the direct effect of altered expression levels on fitness. Indeed, to leading order in the selection coefficients, equations 12–14 in hosts and 17–19 in parasites describe the change in fitness expected if a randomly chosen *m* allele were replaced by an *M* allele within an *Aa* heterozygote.

Examining the signs of equations 12–14 and 17–19 allows us to predict the directions in which expression levels should evolve in heterozygotes. These results are summarized in Figure 1A for the host and Figure 1B for the parasite. As is clear from Figure 1, selection typically favours the evolution of co-expression among hosts but rarely favours co-expression among parasites. These results are conceptually similar to recent findings on the evolution of ploidy levels [18]. In order to recognize and clear a wide array of parasites, selection favours hosts with a broader arsenal of recognition molecules, thus favouring diploid life cycles and the co-expression of alleles in heterozygotes. In contrast, in order to evade a host's immune system or defence response, selection favours parasitic individuals that express a narrow array of antigens and elicitors, thus favouring haploid life cycles or expression of only one allele in heterozygotes. Exceptions to these general rules arise when selection acts in ways other than recognition and evasion. In the MA model, hosts are more likely to survive if they are difficult to mimic, which selects for a narrow expression pattern of only one allele. Furthermore, when costs are added to the GFG model, there are periods of time when selection favours expression of only the least costly allele (i.e., expression of the susceptible allele in hosts when virulence is common among parasites [see equation 12] or the expression of the avirulent allele in parasites when resistance is rare among hosts [see equation 17).

To evaluate whether our analytical results are robust to violations of the assumption that recombination is frequent and selection is weak, we numerically iterated the exact recursions. For each genetic model of co-evolution, we considered both focal hosts and focal parasites, and modifiers that altered the expression probabilities *ρ*_1_, *ρ*_2_, and *ρ*_3_ (Protocol S1). In each case, we considered all combinations of the following selection intensities (0.005, 0.05, and 0.50) and recombination rates (0.005, 0.05, and 0.50) and ran five simulations with randomly chosen initial allele frequencies. In the GFG model, we considered six levels of the costs of expressing only the resistance allele *(C_h_)* or only the virulence allele *(C_p_):* 15%, 30%, 45%, 60%, 75%, or 90% of the value of the fitness cost of infection in hosts, γ_h_, and the fitness cost of resistance in parasites, γ_p_, respectively. In addition, the costs of co-expression *(c_h_* or *c_p_)* were set to 33%, 66%, or 100% of the full costs of resistance or virulence *(C_h_* or *C_p_).* In all simulations, the modifier was introduced at an initial frequency of 0.5 after a 1,000 generation burn-in period had elapsed. All simulations were then run for an additional 4,000 generations, and the modifier was considered to have changed in frequency if its final frequency differed from its initial frequency by an amount greater than 10^−13^. This minimum threshold was set to eliminate false positives due to numerical imprecision and was based upon the maximum change in frequency observed for a modifier with no effect. The simulation results always coincided with the analytical predictions (Figure 1).

**Figure 2:**
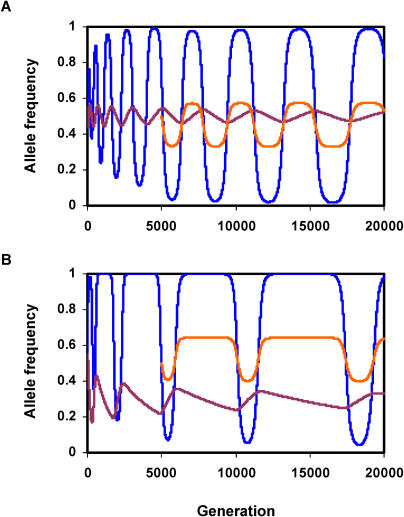
Co-Evolutionary Dynamics of Parasite Gene Expression In both panels, the frequency of a modifier allele that increases the expression of the *B* allele in the parasite population is shown in orange. The frequency of the *B* allele in the parasite population is shown in blue, and the frequency of the *A* allele in the host population is shown in maroon. Both panels considered an expression modifier introduced at an initial frequency of 0.5 with the following effects: *ρ*_1_[*MM*] = 0.75, *ρ*_1_[*Mm*] = 0.50, *ρ*_1_[*mm*] = 0.25, *ρ*_3_[*MM*] = 0.25, *ρ*_3_[*Mm*] = 0.5, *ρ*_3_[*mm*] = 0.75, and *ρ*_2_[*i*] = 1 − *ρ*_1_[*i*] − *ρ*_3_[*i*]. Parameters for the IMA model (A) were α*_h_* = 0.15, α*_p_* = 0.20, *r_h_* = 0.25, and *r_p_* = 0.25. Parameters for the GFG model (B) were γ_h_ = 0.15, γ_p_ = 0.20, *C_h_* = 0.075, *C_p_* = 0.10, *c_h_* = 0.0075, *c_p_* = 0.01, *r_h_* = 0.25, and *r_p_* = 0.25. In both panels, the initial frequency of the *A* allele and the *B* allele was 0.55.

Taken together, our analytical and simulation results suggest that heterozygous hosts should generally evolve to co-express resistance alleles but heterozygous parasites should evolve to express only a single infection allele (Figure 1). It is not clear from the analytical results, however, which allele *(B* or*b),* will ultimately be expressed in heterozygous parasites. Specifically, our analytical results suggest that expression of the *B* allele is favoured at some host allele frequencies, whereas expression of the *b* allele is favoured at others (see equations 17–19). Thus, the potential exists for patterns of parasite gene expression in heterozygotes to cycle over evolutionary time. Results from numerical simulations demonstrate that this is indeed the case. Cycles in parasite gene expression, where allele *B* was expressed during some periods of time and allele *b* at others, were frequently observed in IMA interactions and occasionally in GFG interactions with a cost of resistance (Figure 2). In contrast, cyclical patterns are less likely to persist in host species over long periods of evolutionary time because modifiers that increase co-expression generally spread to fixation (see Figure 1A). Only in the MA model do we expect long-term cycles in levels of dominance to potentially occur in both host and parasite.

## Discussion

Our results demonstrate that co-evolution between hosts and parasites favours co-expression of alleles more often in hosts than in parasites. This predicted pattern is particularly striking among the models with the greatest empirical support (GFG and IMA) and helps explain observed patterns of expression at loci governing infection/resistance in hosts and parasites. Co-expression of resistance alleles has been observed in both the *R* gene family in plant hosts [37,38] and the major histocompatibility complex and immunoglobulin gene families in animal hosts [39]. In contrast, many parasites typically express only one of many antigen alleles encoded by large gene families. For instance, trypanosomes typically express only one of thousands of variant surface glycoprotein genes [40,41]; *Giardia* express only one of 30 to 150 variant-specific surface protein genes [41,42]; ciliates also express only one of many genes encoding surface antigens [43].

Although our modelling framework is quite general in many ways, it makes several important assumptions. First, we have assumed that infection and resistance are mediated by a single genetic locus with only two alleles. Adding additional alleles or loci could conceivably alter our results by changing co-evolutionary dynamics in such a way that polymorphism is either more or less likely to be maintained (e.g., [34]). Because the maintenance of genetic polymorphism is crucial for the evolution of gene-expression modifiers, these effects could be quantitatively important, although we would not expect a qualitative effect. Second, we have not considered limitations on the evolution of increased gene expression that may arise from selection imposed by autoimmune reactions. Increasing the number of parasite-recognition molecules expressed in an IMA or GFG system might increase the likelihood of an autoimmune response. This phenomenon has been demonstrated for the adaptive immune system of vertebrates, where it is thought to select for an intermediate number of antigen receptors [44].

As we have argued, host−parasite interactions provide a theoretical framework in which to understand and interpret the evolution of genetic systems. While we had previously explored the evolution of ploidy levels in hosts and parasites [18], ploidy levels are often relatively stable over evolutionary time and have wide-ranging effects on phenotype beyond their effect on host−parasite interactions [45]. In contrast, expression levels are known to be evolutionarily labile [46] and should be much less constrained by pleiotropy, especially when cis-regulated [47]. As a consequence, we expect the results developed within this paper to yield accurate predictions over a broader range of taxa and types of interactions. Accumulating data on patterns of heterozygous gene expression at loci responsible for infection/resistance will be critical for evaluating this expectation.

## Supporting Information

Protocol S1 Supporting Model Description (85 KB DOC).

## Abbreviations

GFG: gene-for-gene
IMA: inverse-matching-alleles
MA: matching-alleles
