# Supplementary material for: Host–Parasite Interactions and the Evolution of Gene Expression
Source: PLoS Biol. 2005 May 31;3(7):e203. doi: 10.1371/journal.pbio.0030203 (PMC1140679; doi:10.1371/journal.pbio.0030203)
Supplement: Supplementary file 1 [file pbio.0030203.sd001.doc]

# Supporting Model Description

In classical models of the evolution of dominance [e.g., 1,2,3,4,5,6,7] and more recent papers reviewed in Otto and Bourguet [8], the fitness of heterozygotes at a gene **A** is assumed to be under the control of a “modifier” gene, **M**. Modifier alleles that increase heterozygous fitness are favored over those that decrease heterozygous fitness. If variation at the **A** gene is only maintained by mutation, however, the selective force on modifiers of dominance is exceedingly weak (on the order of the mutation rate). This led Wright to conclude that dominance evolution is unlikely to explain the common observation that deleterious mutations are recessive, and he favored metabolic explanations for this phenomenon [3,5]. Wright acknowledged, however, that dominance evolution would be expected in situations where heterozygotes (*Aa*) are common [3, p.277]. Indeed, substantial selection on dominance levels occurs during the spread of beneficial alleles [7,9,10,11,12,13,14,15,16] and at genes with high levels of polymorphism, maintained by frequency-dependent selection [16,17,18,19], overdominant selection [8,20], or migration with spatial variation in selection [8].

Host-parasite interactions present another scenario under which substantial polymorphism can be maintained suggesting that dominance evolution could occur frequently in response to these interactions. In host-parasite interactions, however, there is no standard “wild-type” allele, against which dominance can be measured, as the most fit homozygote changes over time. Thus, to build a model that allowed dominance levels to evolve in a manner that was biologically realistic, we focused on patterns of gene expression in heterozygotes. Our model encompasses the possibility that the heterozygote expresses one allele over the other (as in models of dominance, where evolution favors expression of the wildtype allele), but it also allows for the possibility that evolution might favor co-expression of the alleles in a heterozygote or favor the expression of different alleles at different points in time.

*Quasi-Linkage Equilibrium Approximations*– A full mathematical analysis of (4) is not feasible without making simplifying assumptions. We assume that the interaction coefficients are small in both the host (*γ_h_, ξ_h_, α_h_* <<1) and parasite (*γ_p_, ξ_p_, α_p_* <<1; see Table 1), that costs of resistance and virulence in the gene-for-gene model are small (*C_h_*, *c_h_*, *C_p_*, *c_p_* <<1) when present, and that recombination is frequent relative to the interaction coefficients. Under these assumptions, we can make a quasi-linkage equilibrium (QLE) approximation, where the associations among alleles within a locus (departures from Hardy-Weinberg) and between loci (linkage disequilibria) reach a steady-state level that depends on the current allele frequencies, with this process occurring at a time scale that is fast relative to selection [21,22]. At QLE, we can derive conditions for the spread of a modifier that alters expression levels by calculating the per generation change in the frequency of allele *M*. It is worth pointing out that the following results are not driven by the disequilibrium, which contributes only smaller order terms to changes at the regulatory locus.

*Interpretation of Quasi-Linkage Equilibrium Results* – In the inverse-matching-alleles model, it is always beneficial for the host to recognize and clear a wider variety of parasites. Thus, a modifier that increases the probability of co-expression (Δρ_2,_*_j_* > 0) and decreases the probability of expressing *A* only (Δρ_1,_*_j_* < 0) and/or *a* only (Δρ_3,_*_j_* < 0) is always favored (note negative sign in (7c)), because heterozygous hosts are then able to recognize more parasites. The strength of selection in the hosts, *s*_IMAM,_*_h_*, given by (7c) can be interpreted based on the frequencies of parasites within the population and the fitness interaction table (Table 1). Expressing only *A* as a heterozygous host is disadvantageous to the extent that the parasite expresses only *b* and so cannot be recognized, either because the parasite is homozygous *bb* ($p_{b,p}^{2}$) or because it is heterozygous but expresses only *b* ($2p_{B,p}p_{b,p}\left(\rho_{3,p}\right)$). Thus, increasing expression of *A* instead of co-expressing both alleles in the host ($\Delta\rho_{1,h}$>0) contributes the term $-\alpha_{h}\Delta\rho_{1,h}\left(p_{b,p}^{2}+2p_{B,p}p_{b,p}\left(\rho_{3,p}\right)\right)$ to *s*_IMAM,_*_h_*. Similarly, expressing only *a* as a heterozygous host is disadvantageous to the extent that the parasite expresses only *B*, either because the parasite is homozygous *BB* ($p_{B,p}^{2}$) or is heterozygous and expresses only *B* ($2p_{B,p}p_{b,p}\left(\rho_{1,p}\right)$). Thus, increasing expression of *a* instead of co-expressing both alleles in the host ($\Delta\rho_{3,h}$>0) contributes the term $-\alpha_{h}\Delta\rho_{3,h}\left(p_{B,p}^{2}+2p_{B,p}p_{b,p}\left(\rho_{1,p}\right)\right)$ to *s*_IMAM,_*_h_*. All of the remaining selection terms in (7) and (10) can be understood in a similar manner. In contrast, in the parasite, selection always acts against co-expression, because parasites that express both *B* and *b* can be recognized and cleared by all hosts. Instead, selection favors either expression of allele *B* or *b*, depending on the genotype frequencies within the hosts.

Under the gene-for-gene model without costs, hosts should evolve either co-expression or expression of the resistant allele, *A* (note $-\Delta\rho_{3,h}$ term in (7a)), while parasites should evolve to express only the virulent allele, *B* (note $+\Delta\rho_{1,p}$ term in (10a)). With costs, there is always selection against hosts expressing only the resistant allele, *A*, assuming that their cost of resistance is greater than for hosts co-expressing both alleles (*C_h_* > *c_h_*). Whether co-expression or expression of the sensitive allele, *a*, is favored depends on the allele frequencies, but co-expression should be favored more often when the costs of co-expression are weak. In parasites, costs of virulence select for expression of the avirulent allele, *b*, but unlike in the host, co-expression of both alleles is never favored because co-expressing parasites are no better at infecting resistant hosts than are parasites expressing only *b*.

In the matching-alleles model, selection always acts against co-expression in both hosts and parasites. Co-expression is disadvantageous in hosts because any parasites can mimic a co-expressing host. Co-expression is also disadvantageous in parasites because hosts then have two different alleles that can be recognized as non-self, triggering an immune reaction. Whether expression of allele *A* or *a* (in hosts) or allele *B* or *b* (in parasites) is favored, however, depends on the current frequency of alleles in the interacting species. Selection in hosts favors expression of the allele that cannot be matched by the currently frequent allele in parasites, whereas selection in parasites favors expression of the allele that mimics the currently common allele in hosts.

*Simulations*– Simulations were performed by directly iterating the exact recursions (4). These simulations assume an infinite population size, random mating, and an absence of mutation. For each model of coevolution and combination of parameters (see main text), five replicate simulations were run with initial allele frequencies at the loci under direct selection chosen at random. We considered five cases corresponding to each set of arrows illustrated in Figure 2. For each of these cases we allowed host expression levels to evolve along the arrow and also varied parasite expression levels along the same arrow. In each case, all combinations of s_F_ = {0.005, 0.05, 0.5} and δ_NF_ = {0, 0.25, 0.50, 0.75, 1} were explored. The remaining parameters used are described in the main text.

**Case 1:** Modifier allele *M* increased the probability of expressing *A* (*B*) at the expense of co-expressing *A* (*B*) and *a* (*b*) in the focal species:

| ρ_1,F_[*MM*] = δ_F_ | ρ_1,F_[*Mm*] = δ_F_/2 | ρ_1,F_[*mm*] = 0 |
| --- | --- | --- |
| ρ_2,F_[*MM*] = 1-δ_F_ | ρ_2,F_[*Mm*] = 1-δ_F_/2 | ρ_2,F_[*mm*] = 1 |
| ρ_3,F_[*MM*] = 0 | ρ_3,F_[*Mm*] = 0 | ρ_3,F_[*mm*] = 0 |

In this case, we considered expression patterns in the non-focal species that varied from expression of only *A*(*B*) to complete co-expression of *A* (*B*) and *a* (*b*): ρ_1,NF_ = δ_NF_, ρ_2,NF_ =1-δ_NF_, ρ_3,NF_ = 0.

**Case 2:** Modifier allele *M* increased the probability of expressing *a* (*b*) at the expense of co-expressing *A* (*B*) and *a* (*b*) in the focal species:

| ρ_1,F_[*MM*] = 0 | ρ_1,F_[*Mm*] = 0 | ρ_1,F_[*mm*] = 0 |
| --- | --- | --- |
| ρ_2,F_[*MM*] = 1-δ_F_ | ρ_2,F_[*Mm*] = 1-δ_F_/2 | ρ_2,F_[*mm*] = 1 |
| ρ_3,F_[*MM*] = δ_F_ | ρ_3,F_[*Mm*] = δ_F_/2 | ρ_3,F_[*mm*] = 0 |

In this case, we considered expression patterns in the non-focal species that varied from expression of only *a* (*b*) to co-expression of *A* (*B*) and *a* (*b*): ρ_1,NF_ = 0, ρ_2,NF_ =1-δ_NF_ , ρ_3,NF_ = δ_NF_.

**Case 3:** Modifier allele *M* increased the probability of expressing *A* (*B*) or *a* (*b*) equally often (additive case) at the expense of co-expressing *A* (*B*) and *a* (*b*) in the focal species:

| ρ_1,F_[*MM*] = δ_F_/2 | ρ_1,F_[*Mm*] = δ_F_/4 | ρ_1,F_[*mm*] = 0 |
| --- | --- | --- |
| ρ_2,F_[*MM*] = 1-δ_F_ | ρ_2,F_[*Mm*] = 1-δ_F_/2 | ρ_2,F_[*mm*] = 1 |
| ρ_3,F_[*MM*] = δ_F_/2 | ρ_3,F_[*Mm*] = δ_F_/4 | ρ_3,F_[*mm*] = 0 |

In this case, we considered expression patterns in the non-focal species that varied from expressing *A* (*B*) or *a* (*b*) equally frequently (additive case) to co-expressing *A* (*B*) and *a* (*b*): ρ_1,NF_ = δ_NF_/2, ρ_2,NF_ =1-δ_NF_ , ρ_3,NF_ = δ_NF_/2.

**Case 4:** Modifier allele *M* increased the probability of expressing *A* (*B*) at the expense of expressing *A* (*B*) or *a* (*b*) equally often in the focal species:

| ρ_1,F_[*MM*] =1/2+δ_F_ | ρ_1,F_[*Mm*] = 1/2+δ_F_/2 | ρ_1,F_[*mm*] = 1/2 |
| --- | --- | --- |
| ρ_2,F_[*MM*] = 0 | ρ_2,F_[*Mm*] = 0 | ρ_2,F_[*mm*] = 0 |
| ρ_3,F_[*MM*] = 1/2-δ_F_ | ρ_3,F_[*Mm*] = 1/2-δ_F_/2 | ρ_3,F_[*mm*] =1/2 |

In this case, we considered expression patterns in the non-focal species that varied from expression of only *A* (*B*) to expression of *A* (*B*) or *a* (*b*) with equal probability: ρ_1,NF_ = 1/2+δ_NF_, ρ_2,NF_ = 0 , ρ_3,NF_ = 1/2-δ_NF_.

**Case 5**: Modifier allele *M* increased the probability of expressing *a* (*b*) at the expense of expressing *A* (*B*) or *a* (*b*) equally often in the focal species:

| ρ_1,F_[*MM*] = 1/2-δ_F_ | ρ_1,F_[*Mm*] = 1/2-δ_F_/2 | ρ_1,F_[*mm*] = 1/2 |
| --- | --- | --- |
| ρ_2,F_[*MM*] = 0 | ρ_2,F_[*Mm*] = 0 | ρ_2,F_[*mm*] = 0 |
| ρ_3,F_[*MM*] = 1/2+δ_F_ | ρ_3,F_[*Mm*] = 1/2+δ_F_/2 | ρ_3,F_[*mm*] = 1/2 |

In this case, we considered expression patterns in the non-focal species that varied from expression of only *a* (*b*) to expression of *A* (*B*) or *a* (*b*) with equal probability: ρ_1,NF_ = 1/2-δ_NF_, ρ_2,NF_ =0 , ρ_3,NF_ = 1/2+δ_NF_.

**References**

1. Fisher RA (1928) The possible modification of the response of the wild type to recurrent mutations. American Naturalist 62: 115-126.

2. Fisher RA (1929) The evolution of dominance: reply to Professor Sewall Wright. American Naturalist 63: 553-556.

3. Wright S (1929) The evolution of dominance: comment on Dr. Fisher's reply. American Naturalist 63: 556-561.

4. Wright S (1929) Fisher's theory of dominance. American Naturalist 63: 274-279.

5. Wright S (1934) Physiological and evolutionary theories of dominance. American Naturalist 68: 24-53.

6. Haldane JBS (1930) A note on Fisher's theory of the origin of dominance, and on a correlation between dominance and linkage. American Naturalist 64: 87-90.

7. Haldane JBS (1956) The theory of selection for melanism in Lepidoptera. Proceedings of the Royal Society of London Series B-Biological Sciences 145: 303-306.

8. Otto SP, Bourguet D (1999) Balanced polymorphisms and the evolution of dominance. American Naturalist 153: 561-574.

9. Parsons P, Bodmer WF (1961) The evolution of overdominance in natural populations and heterozygote advantage. Nature 190: 7-12.

10. Bodmer WF (1963) Natural selection for modifiers of heterozygous fitness. Journal of Theoretical Biology 4: 86-97.

11. Ewens W (1966) Linkage and the evolution of dominance. Heredity 21: 363-370.

12. Mayo O (1966) On the evolution of dominance. Heredity 21: 499-511.

13. O'Donald (1967) On the evolution of dominance, overdominance and balanced polymorphism. Proceedings of the Royal Society of London Series B-Biological Sciences 168: 216-228.

14. Wagner GP (1981) Feedback selection and the evolution of modifiers. Acta Biotheoretica 30: 79-120.

15. Burger (1983) Dynamics of the classical genetic model for the evolution of dominance. Mathematical Biosciences 67: 125-143.

16. Wagner GP, Burger R (1985) On the evolution of dominance modifiers. II. A nonequilibrium approach to the evolution of genetic systems. Journal of Theoretical Biology 113: 475-500.

17. Charlesworth D, Charlesworth B (1975) Theortical genetcis of Batesian mimicry. III. The evolution of dominance. Journal of Theoretical Biology 55: 325-337.

18. Clarke CA (1964) Frequency dependent selection for the dominance of rare polymorphic genes. Evolution 18: 364-369.

19. O'Donald P (1968) Models of the evolution of dominance. Proceedings of the Royal Society of London Series B-Biological Sciences 171: 127-143.

20. Feldman MW, Karlin S (1971) The evolution of dominance: a direct approach through the theory of linkage and selection. Theoretical Population Biology 2: 482-492.

21. Nagylaki TJ (1993) The evolution of multilocus systems under weak selection. Genetics 134: 627-647.

22. Kirkpatrick M, Johnson T, Barton N (2002) General models of multilocus evolution. Genetics 161: 1727-1750.
